# Impact of Probiotics, Prebiotics and Synbiotics Supplementation in Chronic Kidney Disease: A Comprehensive Review of Clinical Trials

**DOI:** 10.3390/nu18081176

**Published:** 2026-04-08

**Authors:** Tiziana Di Renzo, Anna Reale, Stefania Nazzaro, Daniela Iovanna, Daniela Evangelista, Vasuk Gautam, Bruna Guida, Rosa Carrano, Mauro Cataldi

**Affiliations:** 1 Institute of Food Sciences, National Research Council, 83100 Avellino, Italy; anna.reale@isa.cnr.it (A.R.); stefania.nazzaro@isa.cnr.it (S.N.); danielaiovanna@cnr.it (D.I.); daniela.evangelista@cnr.it (D.E.); 2 Norton Neuroscience Institute, 200 E. Chestnut St., Louisville, KY 40202, USA; vasuk.gautam@nortonhealthcare.org; 3 Physiology Nutrition Unit, Department of Clinical Medicine and Surgery, University of Naples “Federico II”, 80131 Naples, Italy; bruna.guida@unina.it; 4 Section of Nephrology, Department of Public Health, University of Naples “Federico II”, 80131 Naples, Italy; carrano.rosa5@unina.it; 5 Section of Pharmacology, Department of Neuroscience, Reproductive Sciences and Dentistry, University of Naples “Federico II”, 80131 Naples, Italy; cataldi@unina.it

**Keywords:** microbiota, uremic toxins, gut–kidney axis, Sankey plot, data visualization, precision nutrition

## Abstract

Chronic kidney disease (CKD) is a progressive condition associated with metabolic disturbances, systemic inflammation, and the accumulation of gut-derived uremic toxins. Increasing evidence highlights the role of gut microbiota dysbiosis in the progression of CKD through the gut–kidney axis. Consequently, microbiome-targeted nutritional strategies, including probiotics, prebiotics, and synbiotics, have emerged as promising complementary approaches to modulate intestinal microbial composition and metabolic functions. This review summarizes and critically evaluates the current clinical evidence regarding the use of these interventions in CKD patients. Clinical studies indicate that supplementation with probiotics, prebiotics, and synbiotic formulations may promote beneficial shifts in the composition of the gut microbiota, enhance saccharolytic fermentation, and increase the production of short-chain fatty acids (SCFAs). These changes have been associated with reduced circulating levels of gut-derived uremic toxins such as indoxyl sulfate and *p*-cresyl sulfate, as well as with the attenuation of systemic inflammation and oxidative stress. However, available trials remain heterogeneous in terms of study design, probiotic strains, prebiotic substrates, dosing regimens, and patient populations, and are frequently limited by small sample sizes and short intervention durations. As a result, evidence for improvements in renal function and long-term clinical outcomes remains inconclusive. While synbiotics may offer theoretical advantages by combining microbial supplementation with targeted substrates that support microbial growth and metabolic activity, current evidence does not consistently demonstrate superior clinical efficacy. Overall, these interventions often improve surrogate biomarkers, but their effects on renal function and hard clinical outcomes remain uncertain. Larger, longer-duration multicenter randomized controlled trials with standardized formulations are needed to establish their clinical utility and to better elucidate microbiota–host interactions in CKD. Advancing this field may support the development of personalized microbiome-based therapeutic strategies aimed at modulating the gut–kidney axis and ultimately improving clinical outcomes in CKD patients.

## 1. Introduction

Chronic kidney disease (CKD) is a major global health concern affecting more than 10–13% of adults worldwide [1,2,3,4,5] and is projected to become the fifth leading cause of death by 2040 [6]. It is characterized by progressive deterioration of kidney structure and function and is frequently accompanied by systemic complications. Despite advances in pharmacological therapies and renal replacement treatments, patients with CKD continue to experience a high burden of cardiovascular and immune complications [3], as well as metabolic disorders, chronic inflammation, oxidative stress [7], and alterations in gut microbiota composition [2,4,5,8,9,10,11]. Consequently, current management strategies focus on early detection and the control of modifiable risk factors to delay disease progression and reduce associated complications [12].

Increasing evidence highlights the role of the gut microbiota in CKD pathophysiology. The intestinal microbiota contributes to metabolic, immune, and inflammatory homeostasis, and its disruption has been implicated in several chronic diseases, including renal disorders. The concept of the gut–kidney axis describes the bidirectional interaction between gut microbial communities and kidney function, mediated through immune signaling, inflammatory pathways, intestinal barrier integrity, and microbially derived metabolites. Disturbance of this axis can exacerbate kidney injury and contribute to the progression of CKD and related conditions such as hypertension and diabetes [13].

In healthy individuals, the gut microbiota is mainly dominated by the phyla Firmicutes (including *Clostridium*, *Lactobacillus*, *Bacillus*, *Enterococcus*, and *Ruminococcus*) and Bacteroidetes (primarily *Prevotella* and *Bacteroides*), while Actinobacteria, Proteobacteria, Fusobacteria, and Verrucomicrobia are present at lower relative abundance [14,15].

This balanced ecosystem plays a central role in host–microbe interactions and metabolic regulation. However, the composition of the gastrointestinal tract (GIT) microbiota is highly dynamic and can be influenced by several factors, including antibiotic exposure, immunosuppressant drugs therapy, gastrointestinal infections, psychological and physical stress, radiation, altered intestinal motility, and dietary habits [16,17,18]. Antimicrobial agents, in particular, can profoundly affect microbial communities depending on their spectrum of activity, mechanism of action, pharmacokinetics, dosage, treatment duration, and host-specific factors such as baseline microbiota composition and antimicrobial resistance genes.

CKD is consistently associated with marked alterations in gut microbiota composition and function. Patients commonly exhibit reduced microbial diversity and an increased abundance of bacteria capable of producing uremic toxins. As renal function declines, microbial metabolites such as indoxyl sulfate, *p*-cresyl sulfate, and trimethylamine *N*-oxide (TMAO) accumulate in the circulation, promoting oxidative stress, endothelial dysfunction, systemic inflammation, and further kidney damage. These processes not only accelerate CKD progression but also contribute to the elevated cardiovascular risk observed in this population.

Given the importance of the gut–kidney axis in disease progression, strategies aimed at restoring microbial balance have attracted growing interest. Microbiota-directed approaches, including dietary interventions, prebiotics, probiotics, synbiotics, and postbiotics, seek to modulate microbial composition, reduce the production and absorption of uremic toxins, improve intestinal barrier function, and regulate immune responses [19,20,21,22]. Although several studies have reported beneficial effects of microbiota-targeted interventions in chronic diseases [23,24], clinical evidence in CKD remains heterogeneous and requires further investigation.

This review critically evaluates the impact of probiotic, prebiotic, and synbiotic supplementation in patients with CKD, focusing on clinical studies published between 2015 and 2025. It examines the mechanistic links between gut microbiota alterations and CKD pathophysiology, assesses the clinical outcomes of microbiota-targeted interventions, particularly their effects on renal function and uremic toxin levels, and discusses current limitations and future research directions. It also analyses the temporal trends and the geographical distribution of clinical studies in order to identify gaps in the current literature and highlight areas where research activity has been more extensively conducted. It summarises the available evidence on the potential of these interventions to modulate the composition and activity of the gut microbiota, reduce the production and systemic accumulation of uremic toxins, and influence renal function and metabolic outcomes.

## 2. Materials and Methods

A narrative literature review was conducted to identify clinical studies evaluating microbiota-targeted interventions in patients with chronic kidney disease (CKD). Articles published in English between January 2015 and December 2025 were retrieved from PubMed, Web of Science, and Scopus. The search focused on clinical investigations assessing the effects of probiotics, prebiotics, or synbiotics on CKD-related health outcomes.

After removal of duplicate records, titles and abstracts were screened based on predefined inclusion criteria. Eligible studies included interventional clinical trials and observational clinical investigations involving CKD patients receiving microbiota-modulating interventions. Following this screening process, 55 studies were included in the final analysis. For each study, relevant variables were systematically extracted, including CKD stage, patient characteristics, dialysis status, type of intervention (probiotic, prebiotic, or synbiotic), formulation and dosage, duration of supplementation, biochemical outcomes, and reported changes in faecal microbiota composition.

As this is a narrative review, formal systematic review procedures, including PRISMA flow diagrams, full search strings, dual screening, and structured risk-of-bias assessment, were not applied. Studies were selected and evaluated by the authors for relevance and quality to provide a comprehensive overview of the current clinical evidence.

In addition to summarizing clinical findings, the review explored mechanistic relationships between gut microbiota alterations and CKD pathophysiology, with particular attention to microbial metabolite production, intestinal barrier integrity, and systemic inflammatory processes. To integrate these multidimensional data and visually represent relationships among intervention categories, clinical settings, and biological outcomes, Sankey flow diagrams were employed as analytical and visualization tools. These diagrams were generated using the Python 3.10+ programming language and dedicated visualization libraries, allowing for the organization of extracted variables into categorical nodes connected by directional flows representing reported associations between interventions and outcome domains. The width of each flow was scaled proportionally to the number of studies reporting a given association, thereby reflecting the relative density of available evidence rather than effect size. This approach enabled the simultaneous visualization of multiple dataset dimensions, facilitating comparison across heterogeneous study designs and intervention strategies, while also allowing for the identification of recurring patterns, clusters of evidence, and potential gaps in the literature. For the construction of all Sankey diagrams, outcome variables (including microbiota composition and biochemical/clinical parameters) were categorized a priori as ‘improved’, ‘no change’, or ‘worsened’ based on the overall direction of reported effects at the study level. ‘Improved’ was defined as a statistically significant favorable effect (*p* < 0.05) in at least one primary or clinically relevant outcome, without concurrent evidence of significant adverse effects. ‘No change’ was assigned when studies reported no statistically significant differences or inconsistent findings. ‘Worsened’ was defined as a statistically significant unfavorable effect. When multiple outcomes were reported, classification was based on the predominant direction of clinically relevant results, prioritizing primary endpoints where specified. For crossover designs, only results from the intervention phase compared with the corresponding control condition were considered. Potential carryover effects were taken into account when discussed by the original study authors. This standardized coding approach was applied consistently across all intervention categories to ensure comparability and transparency in the visualization.

All studies included in the Sankey diagrams were classified according to these criteria, regardless of the direction or statistical significance of reported outcomes, to avoid selective reporting bias. All data processing, visualization, and figure generation were performed using Python to ensure methodological transparency and reproducibility.

## 3. Results and Discussion

### 3.1. Evolution and Geographical Distribution of Clinical Studies on Probiotics, Prebiotics, and Synbiotics in CKD

**Figure 1:**
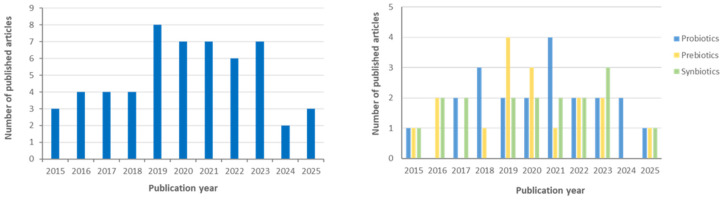
Annual global scientific output of clinical trials investigating probiotics, prebiotics, and synbiotics in patients with CKD from 2015 to 2025 (*n* = 55).

Figure 1 shows the annual global scientific output related to the use of probiotics, prebiotics and synbiotics in clinical trials involving patients with CKD during the period 2015–2025. A total of 55 articles reporting clinical trials were identified, including 21 on probiotics, 17 on prebiotics, and 17 on synbiotics. The publication trend demonstrates sustained research interest over the decade, with a noticeable peak between 2019 and 2023, highlighting the growing attention toward microbiota-based therapeutic strategies in CKD.

**Figure 2:**
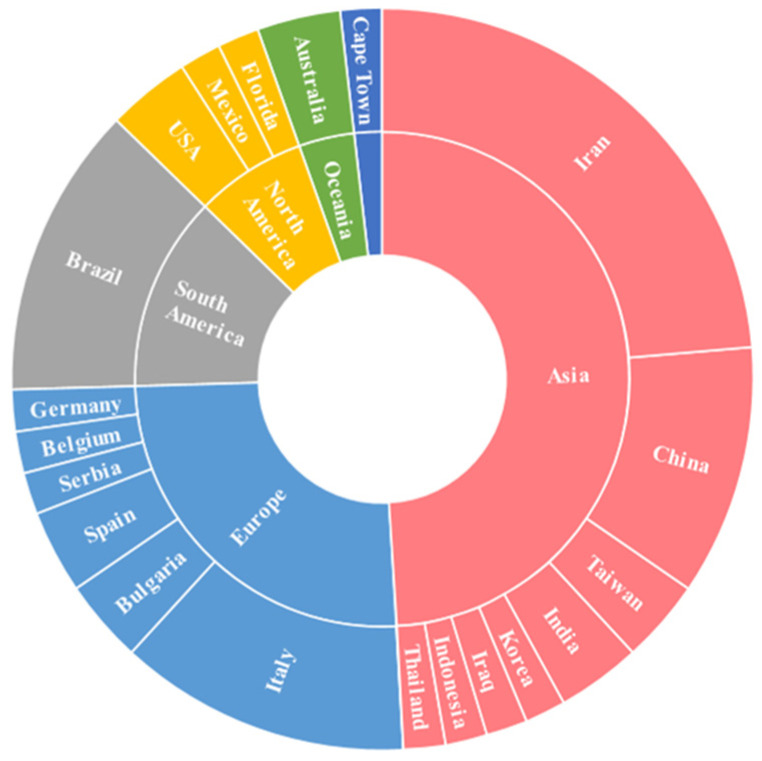
Geographic distribution of clinical trials on probiotics, prebiotics, and synbiotics in patients with CKD from 2015 to 2025.

The geographic distribution of the included studies is shown in Figure 2. Although clinical trials were carried out across several regions worldwide, their distribution was uneven. Nearly half of the studies (49%) originated from Asia, including Iran, China, India, Thailand, Taiwan, Korea, Iraq, and Indonesia. Europe accounted for 25% of the studies, including Italy, Bulgaria, Spain, Serbia, Belgium, and Germany, while the Americas accounted for 20%, with studies conducted in both North and South America. Together, these regions accounted for the majority of clinical research published in the past decade.

**Figure 3:**
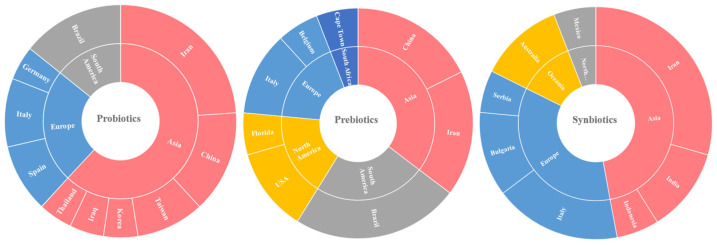
Distribution of clinical trials by type of intervention.

Africa and Oceania contributed a smaller proportion of the studies (5.5%). One clinical trial conducted in Cape Town (South Africa) investigated the use of prebiotics [25], whereas two studies from Australia evaluated synbiotics [26,27]. No probiotic-based clinical trials were identified in these regions. When stratified by intervention type (Figure 3), studies evaluating prebiotics showed the widest geographic distribution, spanning Europe (Italy and Belgium), Asia (China and Iran), the Americas (United States and Brazil), and Africa (South Africa). In contrast, probiotic and synbiotic interventions were more geographically concentrated, with most studies conducted in Europe and Asia. Overall, this global mapping indicates that, between 2015 and 2025, research on microbiota-targeted nutritional interventions in CKD has been concentrated in a limited number of regions, highlighting persistent gaps in global representation.

### 3.2. Use of Probiotics in the Management of CDK in Clinical Studies

**Figure 4:**
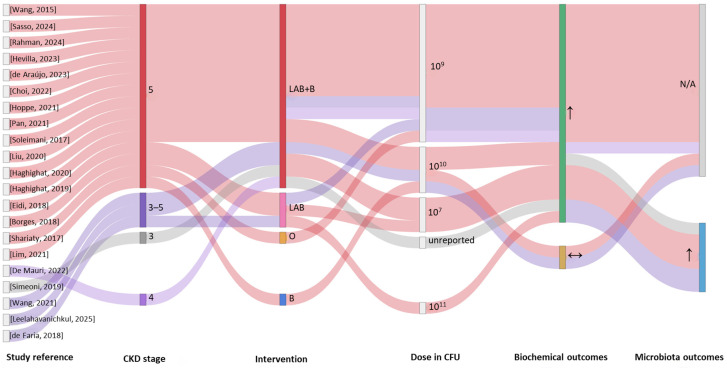
Sankey plot summarizing 21 clinical studies on probiotic supplementation in CKD patients, illustrating CKD stages, probiotic formulations, dosages, microbiota changes, and biochemical outcomes. Wang, 2015 [36], Sasso, 2024 [53], Rahman, 2024 [52], Hevilla, 2023 [51], de Araújo, 2023 [33], Choi, 2022 [50], Hoppe, 2021 [48], Pan, 2021 [46], Soleimani, 2017 [37], Liu, 2020 [45], Haghighat, 2020 [44], Haghighat, 2019 [43], Eidi, 2018 [40], Borges, 2018 [39], Shariaty, 2017 [38], Lim, 2021 [47], De Mauri, 2022 [49], Simeoni, 2019 [42], Wang, 2021 [1], Leelahavanichkul, 2025 [54], de Faria, 2018 [41]. Abbreviations: LAB, lactic acid bacteria; B, *Bifidobacterium* spp.; ↑, increased; ↔, unchanged; N/A, not available.

Probiotics are live microorganisms that, when administered in adequate amounts, confer health benefits to the host [28,29,30]. Not regulated as drugs by the US Food and Drug Administration (FDA) [31], they exert multiple effects, including modulation of the gut microbiota, enhancement of intestinal barrier integrity, and regulation of mucosal function, immunity, inflammation, oxidative stress, host metabolism, and antimicrobial activity [5,32,33,34,35]. In CKD, probiotics have emerged as a promising adjunctive therapy, acting through bacteriocin production, improved intestinal motility, reduced permeability, modulation of innate and adaptive immunity, and regulation of gut-derived uremic toxins and short-chain fatty acids (SCFAs). These mechanisms may alleviate inflammation and oxidative stress and potentially slow CKD progression. Supplementary Table S1 summarizes the main characteristics of 21 clinical trials investigating probiotics across different CKD stages [1,33,36,37,38,39,40,41,42,43,44,45,46,47,48,49,50,51,52,53,54]. Figure 4 presents the results of the Sankey plot derived from 21 clinical studies assessing probiotic supplementation in patients with CKD. Among these, 16 studies involved participants with stage 5 CKD undergoing hemodialysis (HD) or peritoneal dialysis (PD) [33,36,37,38,39,40,43,44,45,46,47,48,50,51,52,53], while the remaining five studies included non-dialysis patients with stage 3–5 CKD [1,41,42,49,54]. Randomized, double-blind, placebo-controlled trials predominated; however, other study designs were also represented, including open-label studies [42,43,44,45,46,47,48], a pilot study [1], a triple-blind trial [40], and non-randomized studies [45,50] (Supplementary Table S1).

Study populations generally consisted of adults over 18 years, most commonly aged 18–80 years, with a mean age of 50–70 years (Supplementary Table S1). This broad age range allowed for inclusion of both younger and older adults, potentially providing insights across age groups. Data on body mass index (BMI) and estimated glomerular filtration rate (eGFR) were reported in only a subset of studies. For those reporting these measures, BMI ranged from 20 to 30 kg/m^2^, indicating participants with normal to slightly overweight status. eGFR values ranged from 10 to 60 mL/min/1.73 m^2^, reflecting varying degrees of kidney function. Sample sizes were small to moderate, ranging from 10 to 116 participants (mean ≈ 47), and intervention durations varied from 4 to 144 weeks, with 12 and 24 weeks being the most common follow-up periods (Supplementary Table S1). The Sankey visualization further illustrates the distribution of studies according to probiotic formulation (Lactic Acid Bacteria [LAB], *Bifidobacterium* [B], combinations [LAB + B], or others [O]), administered dosage, reported microbiota modifications, and biochemical outcomes, providing an integrated overview of the clinical and biological patterns observed across the trials. Outcome classification followed the standardized criteria described in Section 2.

Most interventions (16 studies) employed multi-strain probiotic formulations, predominantly composed of *Bifidobacterium* and *Lactobacillus* species (Figure 4), often combined within the same product. Probiotics were administered orally in various forms, including capsules, sachets, coating powder or bricks (Supplementary Table S1). The number of strains per formulation ranged from a single species in some trials [40,41,42,43,44,45,46,47,48], to two strains of the same species [54], and up to seven strains in others [38,42,55], with multi-species products typically containing three to four strains on average. Probiotic dosages generally ranged from 10^7^ to 10^10^ CFU per day (total CFU per day), administered in one to eight doses daily. When multiple strains were included, the total daily CFU represents the sum of all strains, with individual strain doses indicated when reported. Some protocols included structured “cleaning” or “maintenance” phases to optimize microbial modulation [42]. Overall, the prevailing approach relied on moderate- to high-dose, multi-strain probiotic combinations delivered orally.

Within the *Lactobacillus* genus, the most commonly used species were *Lactobacillus acidophilus*, *Lactiplantibacillus plantarum*, *Lacticaseibacillus rhamnosus*, *Lacticaseibacillus casei*, *Lacticaseibacillus paracasei*, and *Limosilactobacillus reuteri*. For *Bifidobacterium*, the most frequent species were *B. bifidum*, *B. longum*, *B. breve*, and *B. lactis*. Additionally, *Streptococcus thermophilus* was included in three studies [39,46], and *Oxalobacter formigenes* was used in one open-label trial [48] (Supplementary Table S1). All included studies evaluated biochemical outcomes following probiotic therapy, whereas only a limited number (*n* = 5) assessed changes in gut microbiota composition [1,42,45,50,54]. Across randomized controlled and pilot studies, probiotic supplementation was frequently observed with improvements in inflammatory and metabolic profiles. Several trials reported significant reductions in systemic inflammatory markers, including CRP, IL-6, TNF-α, and endotoxemia-related markers, alongside increases in anti-inflammatory mediators such as IL-10. Probiotic interventions also modulated gut-derived uremic toxins, particularly *p*-cresyl sulfate (pCS), indoxyl sulfate (IS), and related metabolites, although effects varied across strains and study designs. These potentially beneficial effects were observed both in non-dialysis patients and in dialysis patients, in whom they were not clearly related to dialysis type. However, comparative studies are still lacking to establish whether dialysis status may affect probiotic efficacy. Renal function indices (eGFR, serum creatinine, blood urea nitrogen [BUN]) generally remained unchanged both in hemodialysis and non-dialysis patients [40,42,52,54] (Supplementary Table S1). However, this lack of effect could be biased by the short duration of most of these studies, considering that long-term observations are critical for evaluating the impact of a treatment on CKD progression. Regarding biochemical outcomes, probiotics were generally associated with improvements in lipid profiles, including reductions in total cholesterol and triglycerides in non-dialysis patients [42,49], and significant decreases in uremic toxins (pCS, IS, phenol) and inflammatory markers (IL-6, IL-5, TNF-α, CRP, hs-CRP) across multiple studies in patients undergoing different dialysis treatments [1,33,36,37,40,42,44,45,46,47,49,50,51,53,54]. In non-dialysis patients, the main reductions observed were in pCS, urinary indicant, urinary and serum IS, CRP, inflammatory markers and BUN [42,49,54].

Collectively, these findings suggest that probiotic supplementation may attenuate systemic inflammation and reduce gut-derived toxin burden, potentially improving cardiovascular and renal outcomes [41,53]. Soleimani et al. [37] reported that in diabetic CKD patients undergoing hemodialysis, probiotics significantly reduced fasting plasma glucose (FPG) (−22.0 vs. +6.6 mg/dL), insulin levels (−6.4 vs. +2.3 mIU/mL), HOMA-IR (−2.9 vs. +2.5), HOMA-B (−14.1 vs. +6.1), glycated hemoglobin (−0.4 vs. −0.1%,), malondialdehyde (MDA) (−0.3 vs. +1.0 mmol/L), and total iron-binding capacity (TIBC), while increasing QUICKI (+0.03 vs. −0.02) and total antioxidant capacity (TAC) (+15 vs. −88 mmol/L), indicating improvements in glycemic control, insulin sensitivity, oxidative stress, and nutritional status.

In non-diabetic CKD patients, probiotics administration restores the gut microbiota and reduces serum levels of pCS, m-cresol, and myo-inositol, thereby helping to improve kidney function and reduce systemic inflammation [45].

These effects may contribute to delaying complications such as protein–energy wasting and vascular disease.

Simeoni et al. [42] examined urinary metabolites of dysbiosis—urinary indican (fermentative dysbiosis marker) and 3-methylindole (3-MI, skatole; putrefactive dysbiosis marker). Both decreased substantially following probiotic therapy, supporting the notion that correcting intestinal dysbiosis reduces microbiota-derived toxins, even in early CKD stages. Hoppe et al. [48] evaluated an oral *Oxalobacter formigenes* formulation in patients with primary hyperoxaluria type 1 (PH1) and end-stage renal disease on dialysis. Long-term supplementation significantly reduced plasma oxalate (Pox) levels at 12 and 24 months and improved left ventricular ejection fraction (LVEF), suggesting that lowering systemic oxalate may mitigate oxalosis-related cardiac dysfunction without increasing dialysis frequency, potentially delaying the need for combined liver–kidney transplantation in advanced PH1. For the purpose of the Sankey visualization, patients with primary hyperoxaluria type 1 undergoing dialysis were categorized within CKD stage 5 dialysis populations, without defining a separate etiological subgroup. Cardiovascular protection was further supported by reductions in endothelial dysfunction markers, including ICAM-1 and syndecan-1, indicating improved endothelial integrity in hemodialysis patients, where vascular damage is often driven by chronic inflammation and uremic toxicity [33,43]. Choi et al. [50] demonstrated immunomodulatory effects: non-classical pro-inflammatory monocytes (CD14^+^CD16^+^) decreased, while regulatory T cells (CD4^+^CD25^+^) increased after three months of probiotic therapy, indicating a shift toward a less pro-atherogenic immune profile.

Gene expression modulation may also contribute to probiotic benefits. Sasso et al. [53] assessed a “Mediterranean diet–like” probiotic-enriched oral nutritional supplement in malnourished hemodialysis patients, focusing on miR-29a and miR-29b, regulators of fibrosis, inflammation, and apoptosis. After three months, both microRNAs were upregulated, with stabilization at six months. Correspondingly, expression of pro-fibrotic and pro-inflammatory genes (RUNX2, TNFA, TGFB1) decreased, whereas PTEN increased. Correlation analyses supported immunomodulatory effects: miR-29a correlated positively with IL-10, and miR-29b inversely with IL-8 and TNF-α. Overall, probiotics in CKD appear to provide multi-level benefits, reducing uremic toxins, attenuating inflammation, improving metabolic and cardiovascular parameters, modulating immune cell profiles, and influencing gene expression, likely via restoration of the gut–kidney axis.

Regarding gut microbiota outcomes, only five of the 21 included trials assessed compositional changes. Probiotic administration generally promoted microbial rebalancing, increasing beneficial taxa such as *Bifidobacterium*, *Lactobacillus*, *Faecalibacterium*, and *Bacteroides* [1,42,45,50,54], while reducing potentially pathogenic groups, including Ruminococcaceae, *Prevotella*, Peptostreptococcaceae, *Collinsella*, Clostridiales Family XIII, and Halomonadaceae [45,50,54]. Effects were more pronounced in non-diabetic subjects [45]. The primary therapeutic aim was to enhance intestinal integrity by restoring commensal bacteria, particularly short-chain fatty acid (SCFA) producers, and reducing toxin-generating taxa. *Bifidobacterium*, in particular, supports epithelial repair and butyrate production through cross-feeding interactions with other beneficial bacteria, such as Lactobacillales. Notably, relative abundance changes sometimes led to decreases in generally beneficial taxa, such as *Faecalibacterium* and *Bacteroides* [50], likely due to competition for substrates and metabolite-mediated shifts in intestinal pH and redox conditions. Across trials, alpha and beta diversity indices (e.g., Chao1, Shannon) were largely unchanged, indicating that probiotics reshaped microbial community structure without consistently increasing overall species richness. It should be noted, however, that these studies were generally underpowered and employed heterogeneous microbiome analysis pipelines (e.g., 16S rRNA amplicon sequencing vs. shotgun metagenomics), which limits direct comparability of diversity metrics across trials.

Overall, probiotic supplementation in CKD appears to exert beneficial immunomodulatory and gut-targeted metabolic effects, particularly in dialysis-dependent populations, though heterogeneity in formulations, dosages, and outcomes limits direct comparability across studies.

### 3.3. Use of Prebiotics in the Management of CKD in Clinical Studies

Prebiotics have gained increasing attention as dietary components capable of modulating the gut microbiota and promoting host health. Since their initial definition in 1995 as non-digestible dietary fibers that selectively stimulate the growth and activity of beneficial bacteria [56], the concept has progressively evolved to encompass a broader range of fermentable substrates and their associated metabolic effects, including SCFAs production and immune modulation. The current definition, established by the International Scientific Association for Probiotics and Prebiotics (ISAPP) in 2017, describes a prebiotic as “a substrate that is selectively utilized by host microorganisms and confers a health benefit” [57], emphasizing selective microbial utilization and the requirement for demonstrated health benefits. Accordingly, prebiotics can be regarded as functional substrates that support beneficial members of the gut microbiota, contributing to intestinal homeostasis and overall host well-being. Today, the main classes of prebiotics include inulin, resistant starch, non-starch polysaccharides (e.g., β-glucans, arabinoxylans, and pectins), fructo-oligosaccharides (FOSs), galacto-oligosaccharides (GOSs), and arabinoxylan-oligosaccharides (AXOSs) [58]. An increasing number of clinical studies suggest that prebiotic supplementation, often combined with a low-protein diet (LPD) or high-fiber dietary regimen, may contribute to slowing CKD progression [59,60]. Between 2015 and 2025, 17 clinical trials in adults across different CKD stages investigated the effects of prebiotics on gut microbiota composition and metabolic outcomes [25,59,60,61,62,63,64,65,66,67,68,69,70,71,72,73,74]. Methodologically, most trials were randomized controlled trials (~80%), frequently adopting double-blind (65%) and placebo-controlled (50%) designs, reflecting efforts toward methodological rigor. Crossover designs were often employed to reduce inter-individual variability in small cohorts, while pilot and open-label trials (~20%) primarily assessed feasibility and tolerability (Supplementary Table S2).

**Figure 5:**
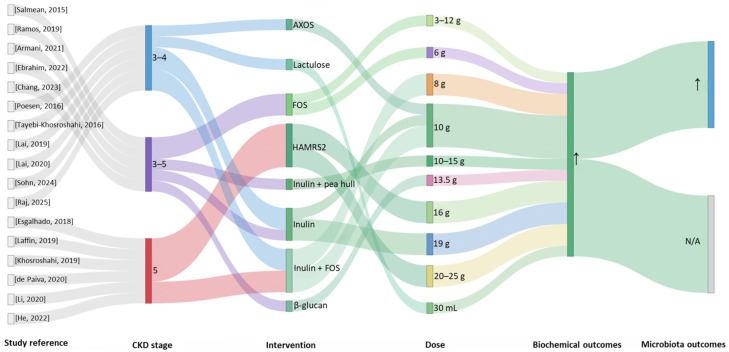
Sankey plot summarizing 17 clinical studies on prebiotic supplementation in CKD patients, illustrating CKD stages, prebiotic types, dosages, microbiota changes, and metabolic outcomes. Salmean, 2015 [61], Ramos, 2019 [65], Armani, 2021 [71], Ebrahim, 2022 [25], Chang, 2023 [60], Poesen, 2016 [62], Tayebi-Khosroshahi, 2016 [63], Lai, 2019 [68], Lai, 2020 [59], Sohn, 2024 [73], Raj, 2025 [74], Esgalhado, 2018 [64], Laffin, 2019 [66], Khosroshahi, 2019 [67], de Paiva, 2020 [69], Li, 2020 [70], He, 2022 [72]. Abbreviations: AXOS, arabinoxylan-oligosaccharides; FOS, fructo-oligosaccharides; HAMRS2, High-amylose maize resistant starch type 2; ↑, increased; N/A, not available.

Figure 5 presents the Sankey plot summarizing 17 clinical studies on prebiotic supplementation in CKD patients.

The plot illustrates the relationships among CKD stage, type of prebiotic compound (including inulin, FOS, AXOS, HAMRS2, lactulose, β-glucan, and combinations), administered dose, and microbiota and metabolic outcomes. Outcome classification followed the standardized criteria described in Section 2. Most studies were conducted in CKD stages 3–5, with a higher representation of stages 3–4 and stage 5 populations.

Inulin, alone or combined with other fiber sources, has been the most commonly studied prebiotic, often administered alongside a low-protein diet (LPD) [59,60,68] or fructo-oligosaccharides (FOSs) [70,72,73,74]. Only one study evaluated inulin combined with pea hull fiber [61]. Four trials assessed high-amylose maize resistant starch type 2 (HAMS2) [64,66,67,69], while two evaluated FOSs alone [65,71]. Other prebiotic sources, such as AXOS, lactulose, and β-glucan, were less commonly studied [25,62,63].

Participants were adults over 18 years, with mean ages ranging from 45 to 70 years, predominantly diagnosed with CKD stages 3–5, frequently stage 5 (Figure 5, Supplementary Table S2). In six of the 17 studies reporting dialysis status, participants were undergoing hemodialysis or peritoneal dialysis [64,66,67,69,70,72]. Sample sizes were generally small, particularly in early or pilot trials (13–21 participants), while more recent studies enrolled 40–59 subjects, with only a few exceeding 50 participants. Intervention durations ranged from 4 to 36 weeks, with most lasting approximately 12 weeks; longer-term studies (24–36 weeks) remain scarce. All interventions were administered orally (one to three times daily), typically in practical formulations such as powders, syrups, muffins, or cookies (Supplementary Table S2). Prebiotic dosages were standardized to 8–25 g per day (g/day), with some liquid preparations administered as approximately 30 mL/day (Figure 5).

In three studies [65,66,67], the doses were titrated to improve gastrointestinal tolerability and adherence, reflecting practical considerations in clinical settings.

Of the 17 clinical studies analyzed, only eight directly evaluated changes in gut microbiota composition following prebiotic supplementation [25,63,66,68,70,72,73,74]. Overall, partial microbial rebalancing was observed, characterized by increases in beneficial taxa, including *Bifidobacterium*, *Lactobacillus*, *Faecalibacterium*, members of Firmicutes, and improvements in the Firmicutes/Bacteroidetes ratio [63,66,72,74], alongside decreases in potentially harmful groups such as Enterobacteriaceae, *Bacteroides*, and Ruminococcaceae [68,70,72,73]. *Faecalibacterium*, a key butyrate-producing genus often depleted in CKD, deserves particular attention. As a strictly anaerobic, oxygen-sensitive bacterium, it is difficult to administer as a probiotic; thus, indirect stimulation through fermentable fibers such as HAMS2 appears more feasible [66]. Its primary metabolite, butyrate, supports intestinal barrier integrity by nourishing colonocytes, strengthening tight junctions, and exerting anti-inflammatory and immunomodulatory effects, including increases in regulatory T cells. Enhanced saccharolytic fermentation may also reduce the generation and systemic absorption of uremic toxins by limiting proteolytic metabolism.

β-glucan supplementation was associated with broader, community-level compositional shifts, modulating genera such as *Prevotella*, *Bacteroides*, *Blautia*, and *Roseburia* [25]. Rather than promoting the expansion of a single genus, β-glucans appeared to gradually restructure the microbial community toward a metabolically favorable configuration, suggesting a modulatory rather than selective amplification effect.

Inulin-based interventions, often administered as inulin-type fructans (ITFs) or oligofructose-enriched inulin, produced more defined species-level changes [70,72,73,74]. These included increases in saccharolytic and potentially purinolytic taxa such as *Anaerostipes* spp., *Clostridium* spp., and members of Clostridiales/Firmicutes, alongside reductions in indole-producing species such as *Bacteroides thetaiotaomicron*. Such remodeling was associated with decreased fecal indole levels and enhanced intestinal uric acid degradation, independent of purine intake or xanthine oxidase activity. These microbial shifts may influence urate handling via purine degradation pathways and SCFA-mediated support of epithelial transport systems (e.g., ABCG2).

Metabolomic analyses demonstrated coordinated shifts across stool, plasma, and urine compartments. While plasma levels of uremic toxins such as *p*-cresol sulfate and indoxyl sulfate did not consistently change, reductions in urinary excretion suggested decreased microbial proteolysis or altered renal handling. Secondary bile acids, including deoxycholic acid, decreased, while carbohydrate fermentation pathways were enriched, consistent with enhanced saccharolytic metabolism. Plasma SCFA levels often remained unchanged, potentially reflecting rapid colonic utilization or peripheral metabolism. Metagenomic sequencing revealed enrichment of saccharolytic species, including *Bifidobacterium longum* and *B. adolescentis*, which are associated with acetate and lactate production, barrier reinforcement, and anti-inflammatory effects [74]. Some taxa, such as *Adlercreutzia equolifaciens* and *Clostridium bartlettii*, increased post-treatment, suggesting delayed or persistent ecological effects, potentially contributing to butyrate production and metabolic regulation, though their roles in CKD remain less well-characterized.

Collectively, these findings indicate that prebiotic supplementation in CKD reshapes microbial metabolic activity rather than restoring global diversity. The most consistent effect is a shift from proteolytic to saccharolytic fermentation, potentially reducing intestinal uremic toxin generation, attenuating systemic inflammation, and supporting gut barrier integrity. Clinical benefits appear highly dependent on disease stage, dialysis status, dietary context, and intervention duration. In advanced CKD, prebiotics are most effective when integrated into stable, well-tolerated dietary frameworks, such as low-protein or fiber-enriched regimens, rather than used in isolation.

Prebiotic supplementation can improve multiple biochemical and metabolic parameters, though effects are fiber-specific and context-dependent (Figure 5, Table S2).

In more than half of the clinical trials examined (9 out of 17), a significant reduction in uremic retention solutes and inflammatory markers was observed. Only three clinical trials reported stable levels of parameters such as pCS and IS following treatment [62,65,70]. Specifically, inulin combined with an LPD consistently reduced protein-bound uremic toxins. In the study of Chang et al. (2023), the median pCS level decreased from 7.52 (2.16–10.75) to 4.02 (1.53–8.43) mg/L, while IS levels declined from 3.42 (2.53–6.01) to 2.83 (1.67–4.74) μg/mL [60].

Regarding inflammatory markers (mean ± SD), Laffin et al. [66] reported that IL-6 decreased from 157.52 ± 83.1 to 118.52 ± 81.6 ng/mL and TNF-α from 318.69 ± 168.7 to 261.26 ± 164.1 ng/mL, whereas in the study of Lai et al. [59], a decrease in CRP concentration from 5.68 ± 3.63 to 3.67 ± 1.88 mg/L was observed. Improvements were also observed in oxidative stress markers (MDA, TBARS, NOX2) [64,66,68], as well as in serum uric acid, phosphorus, sodium, and nitrogen levels [59,60,68,71]. Additionally, benefits included improved acid–base balance, better bowel function, and enhancements in selected quality-of-life domains [61,68]. These interventions also improved glycemic and lipid profiles, reducing glucose, insulin, HOMA-IR, total cholesterol, triglycerides, and homocysteine, while increasing HDL cholesterol [59]. Other fibers, such as AXOS or FOS, were generally safe and well-tolerated but produced limited or selective biochemical effects [62,65,71], potentially due to short intervention durations, lower fermentability, or disease-altered gut environments. High-amylose maize resistant starch type 2 (HAMS2) demonstrated reductions in uremic toxins and inflammatory chemokines (RANTES, PDGF-BB, IP-10), suggesting cardiovascular and renal protective potential [67,69].

Overall, these findings support a model in which prebiotics act as metabolic modulators within the gut–kidney axis: enhancing saccharolytic fermentation, supporting gut barrier integrity, reducing intestinal toxin load, attenuating systemic inflammation, and improving carbohydrate, lipid, and purine metabolism. The magnitude and specificity of effects are fiber- and context-dependent, highlighting the importance of integrating prebiotics into tailored dietary strategies for CKD management. Heterogeneity in study design, small sample sizes, and short intervention durations underscore the need for larger, long-term trials to confirm sustained clinical benefits and optimize therapeutic protocols [68,74].

### 3.4. Use of Complementary Synbiotics in the Management of CDK in Clinical Studies

**Figure 6:**
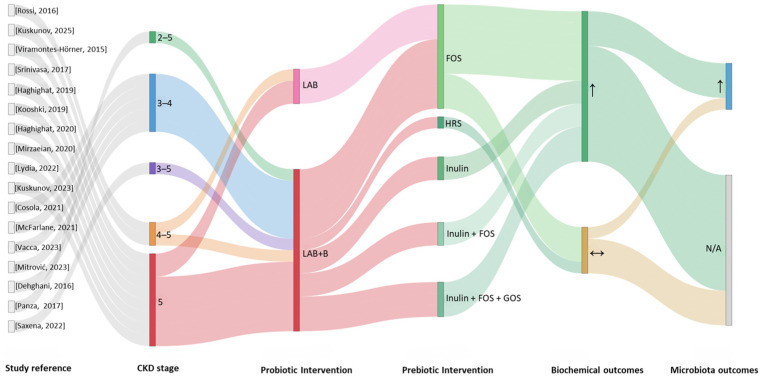
Complementary synbiotic interventions in CKD clinical studies visualized through a Sankey flow diagram. Rossi, 2016 [26], Kuskunov, 2025 [91], Viramontes-Hörner, 2015 [80], Srinivasa, 2017 [82], Haghighat, 2019 [43], Kooshki, 2019 [83], Haghighat, 2020 [44], Mirzaeian, 2020 [84], Lydia, 2022 [87], Kuskunov, 2023 [90], Cosola, 2021 [85], McFarlane, 2021 [27], Vacca, 2023 [88], Mitrović, 2023 [89], Dehghani, 2016 [55], Panza, 2017 [81], Saxena, 2022 [86]. Abbreviations: LAB, lactic acid bacteria; B, *Bifidobacterium* spp.; FOS, fructo-oligosaccharides; HRS, High Resistant Starch; GOS, Galacto-oligosaccharides; ↑, increased; ↔, unchanged; N/A, not available.

In addition to probiotics and prebiotics, synbiotics have been investigated in several clinical studies to modulate the gut microbiota in CKD (Supplementary Table S3). The International Scientific Association for Probiotics and Prebiotics (ISAPP) updated the definition in 2020, describing synbiotics as “a mixture comprising live microorganisms and substrate(s) selectively utilized by the host microorganisms that confers a health benefit to the host” [75]. Each component may confer benefits independently; however, when combined they define a synbiotic formulation. Synbiotics can be synergistic, where the substrate specifically supports the co-administered microorganisms, or complementary, where the probiotic is paired with a substrate targeting the indigenous microbiota. Prebiotics support probiotic activity through several mechanisms: enhancing probiotic stability during storage and gastrointestinal transit, serving as substrates that promote microbial proliferation and colonization, and indirectly shaping the gut microbial ecosystem to create favorable ecological conditions, which may vary according to individual microbiota profiles [76,77,78,79]. Consequently, combining prebiotics and probiotics may provide greater potential for modulating gut microbiota and slowing CKD progression. Figure 6 summarizes the results of 17 clinical studies evaluating complementary synbiotic supplementation in CKD using a Sankey flow diagram [26,27,43,44,55,80,81,82,83,84,85,86,87,88,89,90,91]. Outcome classification followed the standardized criteria described in Section 2.

The visualization integrates information on CKD stage, probiotic composition (Lactic acid bacteria alone [LAB] or L + *Bifidobacterium* [LAB + B]), associated prebiotic compounds (e.g., FOS, inulin, HRS, GOS, or combinations), administered doses, microbiota outcomes, and metabolic responses. Most studies enrolled patients with CKD stages 3–5, with stage 5 prominently represented. Combined probiotic preparations (LAB + B) were more frequently used than single *Lactobacillus* strains. The most common prebiotic components were FOS and inulin, administered either alone or with other fermentable fibers [26,43,44,55,80,81,82,83,84,85,86,87,88,89,90,91]. Prebiotic doses varied widely (approximately 0.065–20 g/day), whereas probiotic doses most often ranged between 10^9^ and 10^11^ CFU/day. Microbiome modulation was reported in several studies, although some trials did not evaluate microbial outcomes [26,27,88,89]. In contrast, biochemical parameters were more consistently assessed and were generally described as improved, with fewer studies reporting no significant changes. Overall, the diagram highlights the diversity of complementary synbiotic formulations and dosing strategies across CKD stages, alongside a general predominance of favorable biochemical outcomes. The probiotic strains most frequently used included *Lactobacillus acidophilus*, *Lacticaseibacillus casei*, *Lacticaseibacillus paracasei*, *Bifidobacterium longum*, *Bifidobacterium lactis*, *Bifidobacterium breve*, *Bifidobacterium animalis*, and *Streptococcus thermophilus*, typically administered orally as gels, tablets, capsules, or powders (Supplementary Table S3) at doses ranging from 10^7^ to 10^11^ CFU/day. The most common prebiotic substrates were inulin, resistant starch, fructooligosaccharides (FOSs), and galactooligosaccharides (GOSs), used either individually [27,55,80,81,82,83,84,86,87,89,90,91] or in combination [26,43,44,85,88], with doses ranging from 0.1 to 20 g/day. Combined synbiotic doses generally ranged from 0.1 to 2.5 g/day and were often initiated at lower levels to improve tolerability. Most trials (12/17) were randomized, double-blind, and placebo-controlled, while others were crossover, open-label [82], or quasi-experimental [90]. Participants were adults aged 30–90 years with CKD stages 2–5, including eight studies with stage 5 patients (Figure 6), many undergoing hemodialysis [43,44,80,82,83,84,87,90,91]. Sample sizes ranged from 23 to 85 participants (average ≈ 46), and intervention durations varied from 6 to 48 weeks, most commonly 8–12 weeks (Supplementary Table S3).

Clinical outcomes were assessed according to recommendations from the Standardized Outcomes in Nephrology (SONG) initiative, including renal function markers (eGFR, creatinine, albuminuria, proteinuria), uremic toxins (urea, IS, pCS, TMAO), gastrointestinal outcomes (microbiota composition, fecal metabolites, and symptoms), and inflammatory and oxidative stress markers (IL-6, hs-CRP, TNF-α, malondialdehyde [MDA], and heat shock proteins [HSPs]). Overall, most studies involving non-dialysis patients reported reductions in uremic toxins, inflammatory markers, and oxidative stress [26,83,85,86,89], with stable or slightly improved eGFR in some cases [27,86,89]. Gastrointestinal symptom outcomes were less consistent [80,85]. Only two studies [81,84] observed transient increases in uremic toxins or parathyroid hormone (PTH), possibly related to short intervention periods, formulation differences, dietary intake, or hemodialysis-related factors. Current dialysis techniques effectively remove small water-soluble uremic toxins, whereas middle-molecular-weight toxins are only partially cleared. In contrast, protein-bound uremic toxins are poorly eliminated due to their strong affinity for albumin, which limits their dialytic removal [84]. To minimize such variability in future trials, a stabilized dietary run-in period prior to intervention initiation is recommended. Furthermore, as proposed by Panza et al. [81], synbiotic interventions should be initiated during the early stages of CKD and sustained over prolonged periods.

In some trials, proteolytic enzymes were added to complementary synbiotic preparations (enzobiotics), improving protein metabolism, serum albumin levels, and hematological parameters such as red blood cell and platelet counts, factors often compromised in CKD, and potentially contributing to slower disease progression [86]. Among the analyzed studies, only four specifically assessed gut microbiota changes after complementary synbiotic supplementation. These reported a shift toward a more balanced microbial profile, with increases in beneficial taxa such as *Bifidobacterium*, *Lachnospiraceae*, *Faecalibacterium*, and *Blautia*, along with a higher Firmicutes/Bacteroidetes ratio. At the same time, taxa associated with dysbiosis, including *Clostridiales*, *Ruminococcaceae*, and *Flavobacteriaceae*, tended to decrease. Such changes suggest reduced proteolytic fermentation and lower production of gut-derived uremic toxins.

Complementary synbiotic supplementation therefore appears to promote a SCFA-producing microbiota (*Bifidobacterium* spp. and *Lachnospiraceae*) [26,27,88], reducing inflammation (CRP) [89] and supporting the gut–kidney axis as a complementary therapeutic approach in stages 3–5 CKD [26,27,88,89]. Faecal metabolomic analyses further demonstrated increased short-chain fatty acids, particularly acetic and propionic acids, reflecting enhanced saccharolytic fermentation and shifts in microbial composition, while decreasing potentially harmful sulfur metabolites [88]. Two studies identified molecular mechanisms that could account for beneficial effects of symbiotic supplementation on cardiovascular risk [43,44]. In the first one, the prespecified primary outcome was an absolute change in the adhesion proteins ICAM-1 or VCAM-1. After adjustment for confounders, only ICAM-1 was significantly lower in the symbiotic group compared with the control group [43]. The primary endpoint of the second study was a change in anti-HSP70 antibodies, which have been linked to endothelial damage and cardiovascular diseases [44]. Overall, these findings support the potential role of complementary synbiotics in improving intestinal metabolism, reducing uremic toxin burden, and mitigating cardiovascular and inflammatory complications associated with CKD.

In conclusion, current clinical evidence indicates that complementary synbiotic supplementation in CKD patients, particularly in stages 3–5, can beneficially modulate gut microbiota, increase SCFA production, reduce uremic toxins, and attenuate systemic inflammation and oxidative stress. Although effects on renal function and gastrointestinal symptoms remain variable, these interventions, alone or combined with proteolytic enzymes, show promise for improving protein metabolism, cardiovascular parameters, and markers of gut–kidney axis integrity. Larger and longer-term clinical trials are still needed to optimize formulations, dosages, and intervention durations to maximize clinical benefits [26,27,43,44,88,89].

## 4. Conclusions and Future Perspectives

Current evidence indicates that microbiome-targeted nutritional interventions, including probiotics, prebiotics, and synbiotics, can positively influence the gut ecosystem in patients with CKD. Clinical studies suggest that these approaches may enhance short-chain fatty acid (SCFA) production, reduce the generation and systemic accumulation of gut-derived uremic toxins, and attenuate inflammatory and oxidative pathways implicated in CKD progression. Collectively, these findings support the relevance of the gut–kidney axis as a therapeutic target and highlight the potential role of microbiota modulation as a complementary strategy in CKD management.

However, despite encouraging results, the overall strength of the evidence remains moderate and should be interpreted cautiously. Most available trials are characterized by relatively small sample sizes, short intervention periods, and substantial heterogeneity in study design, patient populations, probiotic strains, prebiotic substrates, and dosing regimens. These methodological differences complicate direct comparisons between studies and limit the ability to draw definitive conclusions regarding efficacy, optimal formulations, and long-term clinical benefits. Furthermore, many studies focus primarily on surrogate biochemical markers, such as uremic toxins or inflammatory mediators, rather than clinically meaningful endpoints, including CKD progression, cardiovascular events, hospitalization, or mortality.

Of the evaluated strategies, synbiotic formulations are of interest because they combine beneficial microorganisms with substrates that support their growth and metabolic activity. Therefore, they are expected to have a synergistic effect. Several studies suggest that synbiotics could help promote microbial diversity, support beneficial taxa involved in saccharolytic fermentation, and potentially enhance SCFA production, thereby contributing to improved intestinal barrier function and reduced systemic inflammation. However, due to the lack of comparative studies, it is unclear whether synbiotics offer any advantages over probiotic preparations. Furthermore, the heterogeneity of synbiotic formulations and the limited number of trials specifically assessing microbiota composition or functional changes remain significant limitations. In addition, considerable inter-individual variability in treatment response has been observed, likely reflecting differences in baseline microbiota composition, CKD stage, comorbidities, medication use, and dietary patterns. These factors underscore the importance of adopting personalized microbiota-based nutritional interventions. Future research should therefore prioritize large-scale, multicenter randomized controlled trials with longer follow-up periods to evaluate the sustained effects of microbiota-targeted interventions on both biological markers and clinically relevant outcomes. Standardization of probiotic strains, prebiotic substrates, and dosing protocols will be essential to enable meaningful comparisons across studies and to identify the most effective therapeutic combinations. At the same time, mechanistic investigations integrating multi-omics approaches, including metagenomics, metabolomics, transcriptomics, and proteomics, will be crucial for elucidating the microbial pathways, metabolites, and host signaling networks that mediate interactions within the gut–kidney axis. Another important research direction involves the integration of microbiome-targeted therapies with established nutritional strategies for CKD, such as low-protein or fiber-enriched diets, which may synergistically influence microbial metabolism and reduce the production of uremic toxins. Advances in delivery technologies, including encapsulation systems or targeted microbial consortia, may further improve probiotic viability, intestinal colonization, and therapeutic efficacy. Additionally, expanding research to underrepresented populations, such as pediatric patients, elderly individuals, and patients undergoing dialysis, will help clarify age- and stage-specific responses to these interventions. Overall, while microbiome-based therapies represent a promising and biologically plausible adjunct in CKD management, their translation into routine clinical practice requires stronger clinical evidence and a deeper understanding of host–microbiome interactions. Bridging clinical research with system biology, microbiome science, and nutritional therapeutics may ultimately enable the development of precision microbiome–based strategies capable of modulating the gut–kidney axis and improving long–term outcomes in patients with CKD.

Key message

(1) What is known
  - Gut microbiota dysbiosis contributes to CKD progression via the gut–kidney axis;
  - Microbiota-targeted interventions, including probiotics, prebiotics, and complementary synbiotics, can modulate microbial composition and metabolic activity.
(2) What this review adds
  - Summarizes clinical trial evidence on biochemical, functional, and limited clinical outcomes of microbiota-targeted interventions in CKD;
  - Highlights variability in study design, populations, and endpoints, emphasizing functional outcomes such as SCFA production.
(3) Clinical implications
  - Interventions are generally safe and well-tolerated, but effects on renal function and long-term clinical outcomes remain uncertain;
  - Personalized approaches considering baseline microbiota, CKD stage, comorbidities, and diet may optimize response.
(4) Research priorities
  - Conduct large-scale, multicenter RCTs with ≥6–12 months follow-up, standardized formulations, and consistent dosing;
  - Include SONG CKD core outcomes and functional microbiome readouts (SCFAs, metabolites) to enhance translational relevance;
  - Integrate multi-omics approaches to elucidate gut–kidney axis mechanisms.

## Data Availability

The original data presented in the study are openly available in GitHub repository: https://github.com/VasuRAD/Plotly_SankeyPlot/tree/main (accessed on 3 April 2026) and Read me: https://github.com/VasuRAD/Plotly_SankeyPlot/blob/main/README.md (accessed on 3 April 2026).

## Supplementary Materials

The following supporting information can be downloaded at https://www.mdpi.com/article/10.3390/nu18081176/s1, Table S1: Key characteristics and findings of clinical studies investigating the use of probiotics in patients with chronic kidney disease (CKD); Table S2: Key characteristics and findings of clinical studies investigating the use of prebiotics in patients with chronic kidney disease (CKD); Table S3: Key characteristics and findings of clinical studies investigating the use of synbiotics in patients with chronic kidney disease (CKD).
