# Supplementary material for: Impact of Probiotics, Prebiotics and Synbiotics Supplementation in Chronic Kidney Disease: A Comprehensive Review of Clinical Trials
Source: Nutrients. 2026 Apr 8;18(8):1176. doi: 10.3390/nu18081176 (PMC13118840; doi:10.3390/nu18081176)
Supplement: Supplementary file 1 [file nutrients-18-01176-s001.zip › nutrients-4229100-supplementary/Supplementary Table S1.pdf]

Supplementary Table S1. Key characteristics and findings of clinical studies investigating the use of probiotics in patients with chronic kidney disease (CKD).

Probiotic composition	Probiotic dose	Study design	Patient characteristics	Sample size	CKD stage	Dyalisis Status	Route of Administration Intervention duration	Biochemical outcomes	Microbiota outcome	Origin	Reference
Bifidobacterium bifidum A218, *Bifidobacterium catenulatum* A302, *Bifidobacterium longum* A101, *Lactiplantibacillus plantarum* A87	10^9 CFU/capsule	Placebo-controlled, parallel-group RCT	≥18 years	39	5	PD	**Administration**: oral (1 capsule/day) **Duration**: 24 weeks	↓ TNF-α, IL-6, IL-5, endotoxemia ↑ IL-10	N/A	Asia (Taiwan)	[36]
Lactobacillus acidophilus, Lacticaseibacillus casei, Bifidobacterium bifidum	2 x 10^9 CFU/capsule	Placebo-controlled RCT	18–80 years	60	5	H	**Administration**: oral (1 capsule/day) **Duration**: 12 weeks	↓ FPG, insulin, HOMA-IR, MDA, HbA1c, hs-CRP ↑ QUICKI, TAC	N/A	Asia (Iran)	[37]
Lactobacillus acidophilus, Lacticaseibacillus casei, Lacticaseibacillus rhamnosus, Lactobacillus delbrueckii subsp. *bulgaricus* , *Bifidobacterium breve, Bifidobacterium longum, Streptococcus thermophilus*	3 x 10^{10} CFU/capsule	Double-blind, placebo-controlled RCT	>17 years	36	5	H	**Administration**: oral (1 capsule/day) **Duration**: 12 weeks	↑ Hb	N/A	Asia (Iran)	[38]
Streptococcus thermophilus, Lactobacillus acidophilus, Bifidobacterium longum	3 x 10^{10} CFU/capsule	Double-blind, placebo-controlled RCT	≥18 years	46	5	H	**Administration**: oral (3 capsules/day) **Duration**: 12 weeks	↑ pre-BUN, potassium, IS ↔ p-CS, IAA	N/A	South America (Brazil)	[39]
Lacticaseibacillus rhamnosus	1.6 x 10^7 CFU/capsule	Triple-blind, placebo-controlled RCT	>20 years	42	5	H	**Administration**: oral (1 capsule/day) **Duration**: 4 weeks	↓↓ p-CS, phenol ↔ electrolytes, BUN, Cr	N/A	Asia (Iran)	[40]
Lactobacillus acidophilus, Lacticaseibacillus casei, Bifidobacterium bifidum	3 x 10^{10} CFU/capsule	Double-blind, placebo-controlled RCT	57-71 years; BMI of 27 ± 4 kg/m^2, eGFR: 34 ± 9 mL/min/1.73 m^2	30	3–5		**Administration**: oral (3 capsules/day) **Duration**: 12 weeks	↑ IL-6 ↔ routine biochemical parameters, IS, p-CS, TMAO, choline, betaine, CRP, LPS, calprotectin	N/A	South America (Brazil)	[41]

Enterelle® (*Enterococcus faecium, Lactobacillus acidophilus, Saccharomyces boulardii*); Bifiselle® (*Bifidobacterium brevis, Bifidobacterium bifidum, Bifidobacterium longum*); Ramnoselle® *(Lacticaseibacillus rhamnosus, Lactobacillus acidophilus*)	N/A	Open-label, placebo-controlled	>18 years; eGFR: 45–60 ml/min/1.73 m^2	28	3		**Administration**: oral<br>•3 capsules/day - Enterelle®: 1 week<br>•3 capsules/day Bifiselle®+Ramnoselle®): 2 weeks<br>•2 capsules/day of Bifiselle®+Ramnoselle®): 12 weeks<br>**Duration**: 15 weeks	↓ urinary indican, 3-Methylindole, ILA, urinary IS and pCS, CRP, TC, TG, B2M, ferritin<br>↑ serum calcium and iron, TSAT<br>↔ PTH, eGFR	↑Lactobacillales, *Bifidobacterium* spp	Europe (Italy)	[42]
Lactobacillus acidophilus, Bifidobacterium bifidum, Bifidobacterium lactis, Bifidobacterium longum	2.7 x 10^7 CFU/g each	Double-blind, placebo-controlled RCT	30-65 years	50	5	H	**Administration**: oral (5 g probiotic powder 4 times/day)<br>**Duration**: 12 weeks	↓ ICAM-1<br>↔ CK-18, UA, phosphate	N/A	Asia (Iran)	[43]
Lactobacillus acidophilus, Bifidobacterium bifidum, Bifidobacterium lactis, Bifidobacterium longum	2.7 x 10^7 CFU/g each	Double-blind, placebo-controlled RCT	30-65 years	50	5	H	**Administration**: oral (5 g probiotic powder 4 times/day)<br>**Duration**: 12 weeks	↓↓ anti-HSP70<br>↓ IL-6	N/A	Asia (Iran)	[44]
Enterococcus faecalis, Bifidobacterium longum, Lactobacillus acidophilus	2.2 x 10^9 CFU/capsule	Double-blind, placebo-controlled	18–70 years	50	5	H	**Administration**: oral (4 capsules twice daily)<br>**Duration**: 24 weeks	↓ IAA-O-glucuronide (serum), 1-methylinosine (serum and feces), 3-GPA and phenol (feces)<br>In non-diabetic patients undergoing hemodialysis:<br>↓ pCS, m-cresol, myoinositol	In non-diabetic patients undergoing hemodialysis:<br>↓Ruminococcaceae, Peptostreptococcaceae,Erysipelotrichaceae ↓Clostridiales Family XIII, Halomonadaceae<br>↑Bacteroidaceae, Enterococcaceae	Asia (China)	[45]
Bifidobacterium longum, Lactobacillus delbrueckii subsp. *bulgaricus, Streptococcus thermophilus*	1 x 10^9 CFU/capsule	Placebo-controlled, parallel-group RCT	18–75 years	116	5	CPD	**Administration**: oral (2 capsules/3 times/day)<br>**Duration**: 8 weeks	↓hs-CRP, IL-6, LDL-C, TG<br>↔ albumin	N/A	Asia (China)	[46]

Lactococcus lactis<br>*Lactobaccillus salivarius*<br>*Lactobaccillus pentosus*	10^{11} CFU/capsule	Double-blind, placebo-controlled RCT	39–75 years	56	5	H	**Administration**: oral (1 capsule twice daily)<br>**Duration**: 24 weeks	↓ IS	N/A	Asia (Taiwan)	[47]
Bifidobacterium bifidum<br>*Lactobacillus acidophilus*<br>*Bifidobacterium longum*	2.5 x 10^9 CFU/capsule	Single-arm, pilot	≥20 years; eGFR: 30 ± 16 mL/min/1.73m^2	44	3-5		**Administration**: oral (2 capsules/day)<br>**Duration**: 24 weeks	↓ eGFR, IL-6, IL-18, TNF-α<br>↔ BP	↑*Bifidobacterium bifidum, Bifidobacterium breve*<br>*Faecalibacterium* (dominant genus)	Asia (China)	[1]
Oxalobacter formigenes	>10^9 CFU/capsule	Open-label	20-58 years; BMI: 20-32 kg/m^2	12	5	H	**Administration**: oral (1 capsule/day)<br>**Duration**: 144 weeks	↓ fPOX, tPOX	N/A	Europe (Germany)	[48]
Bifidobacterium longum, Limosilactobacillus reuteri (LRE02)	5 x 10^9 CFU/dose	Double-blind, single-centre, placebo-controlled RCT	18–80 years; eGFR: <25 mL/min/1.73m^2	60	4		**Administration**: oral (two 2-g dose/day for 4 weeks, then one 2-g dose/day for 8 weeks)<br>**Duration**: 12 weeks	↓ BUN, TC, TG, TUN, PCR, nPCR, IS, pCS, Lp-PLA2<br>↔ urine protein excretion, LDL-C	N/A	Europe (Italy)	[49]
Bifidobacterium bifidum BGN4, *Bifidobacterium longum* BORI	7 x 10^9 CFU/g	Non-RCT	≥18 years; BMI: 21-25 kg/m^2	22	5	H	**Administration**: oral (2-g sachets twice daily)<br>**Duration**: 12 weeks	↓ sCP, IL-6, pro-inflammatory $CD14^+CD16^+$ monocytes<br>↑ SCFA, $CD4^+CD25^+$ regulatory T cells<br>↔ CRP, albumin, calcium, phosphate, PTH	↓ *Bacteroides, Faecalibacterium, Tyzzerella, Sutterell*[illegible] *Akkermansia* genera<br>↓ *Eubacterium siraeum*<br>↑ *Prevotella, Enterococcus, Alistipes, Clostridia, Escherichia-Shigella Klebsiella, Bifidobacterium* genera	Asia (Korea)	[50]
Lactiplantibacillus plantarum A87, *Lacticaseibacillus rhamnosus*, *Bifidobacterium bifidum* A218, *Bifidobacterium longum* A101	4 x 10^9 CFU/capsule	Double-blind, placebo-controlled RCT	22–69 years	70	5	H	**Administration**: oral (1 capsule/day)<br>**Duration**:12 weeks	↓ CRP, syndecan-1, glucose<br>↑ Hb, Hct	N/A	South America (Brazil)	[33]

Bifidobacterium breve CNCM I-4035, *Bifidobacterium animalis lactis* BPL1 CECT 8145, *Lacticaseibacillus paracasei* CNCM I-4034	3.5 x 10^9 CFU/capsule	Double-blind, parallel-group RCT	Malnourished patients (65-84 years)	59	5	H	**Administration**: oral (1 capsule/day + 2 bricks of 400 mL/day of ONS) **Duration**: 24 weeks	↓ MCP-1, IL-1β, IL-4, IL-8, BMP-2, IL-10, potassium ↑ vitamin D, prealbumin, TAC	N/A	Europe (Spain)	[51]
Lactobacillus acidophilus, Bifidobacterium bifidum	2 x 10^9 CFU/capsule	RCT	18–75 years	42	5	H	**Administration**: oral (1 capsule/day) **Duration**: 8 weeks	↓ urea, phosphorus ↔ Cr, eGFR, albumin, IS, BP	N/A	Asia (Iraq)	[52]
Bifidobacterium breve CNCM I-4035, *Bifidobacterium animalis lactis* BPL1 CECT 8145, *Lacticaseibacillus paracasei* CNCM I-4034	3.5 x 10^9 CFU/capsule	Double-blind, parallel-group RCT	Malnourished patients (65-84 years)	59	5	H	**Administration**: oral (1 capsule/day + 2 bricks of 400 mL/day of ONS) **Duration**: 24 weeks	↓ TNF-α, RUNX2, TGF-β1 genes ↑ PTEN, miR-29a and miR-29b expression	N/A	Europe (Spain)	[53]
Lacticaseibacillus rhamnosus L34/*Lacticaseibacillus rhamnosus* GG	3.5 x 10^9 CFU/powder	Placebo-controlled RCT	≥18 years; eGFR: 10–60 mL/min/1.73 m^2	75	3-5		**Administration**: oral (coating powder/day) **Duration**: 4 weeks	↓ fIS, pCS, TNF-α, IL-6, IL-10, NETs ↔ Cr, BUN	↓ Bacteroidota *(Bacteroides* spp.) ↑ Actinobacteria *(Collinsella* spp.)	Asia (Thailand)	[54]

Abbreviations: RCT, randomized controlled trial; PD, peritoneal dialysis; H, hemodialysis; CPD, chronic peritoneal dialysis; TNF-α, tumor necrosis factor-alpha; IL-5, interleukin-5; IL-6, interleukin-6; IL-10, interleukin-10; FPG, fasting plasma glucose; HOMA-IR, homeostatic model assessment for insulin resistance; MDA, malondialdehyde; HbA1c, hemoglobin A1C; hs-CRP, high-sensitivity C-reactive protein; QUICKI, quantitative insulin sensitivity check index; TAC, total antioxidant capacity; Hb, hemoglobin; pre-BUN, pre-dialysis blood urea nitrogen; IS, indoxyl sulfate; p-CS, p-cresyl sulfate; IAA, indoleacetic acid; BUN, blood urea nitrogen; Cr, creatinine; CRP, C-reactive protein; LPS, lipopolysaccharide; TMAO, trimethylamine N-oxide; ILA, indole-3-lactic acid; TC, total cholesterol; TG, triglycerides; B2M, beta-2-microglobulin; TSAT, transferrin saturation; PTH, parathyroid hormone; eGFR, estimated glomerular filtration rate; ICAM-1, intercellular adhesion molecule-1; CK-18, cytokeratin-18; UA, uric acid; anti-HSP70, anti-heat shock protein 70; IAA-O-glucuronide, indole-3-acetic acid O-glucuronide; 3-GPA, 3-guanidinopropionic acid; LDL-C, low-density lipoprotein cholesterol; BP, blood pressure; fPOX, free plasma oxalate; tPOX, total plasma oxalate; TUN, total urinary nitrogen; PCR, protein catabolic rate; nPCR, normalized protein catabolic rate; Lp-PLA2, lipoprotein-associated phospholipase A2; SCFA, short chain fatty acids; Hct, hematocrit; MCP-1, monocyte chemoattractant protein-1; IL-1β, interleukin-1 beta; IL-4, interleukin-4; IL-8, interleukin-8; IL-18, interleukin-18; BMP-2, bone morphogenetic protein 2; RUNX2, runt-related transcription factor 2; TGF-β1, transforming grow factor-beta1; PTEN, phosphatase and tensin homolog; fIS, free indoxyl sulfate; NET, neutrophil extracellular traps; ↓, decreased; ↑, increased; ↔, unchanged; N/A, not available.
