# Supplementary material for: Impact of Probiotics, Prebiotics and Synbiotics Supplementation in Chronic Kidney Disease: A Comprehensive Review of Clinical Trials
Source: Nutrients. 2026 Apr 8;18(8):1176. doi: 10.3390/nu18081176 (PMC13118840; doi:10.3390/nu18081176)
Supplement: Supplementary file 1 [file nutrients-18-01176-s001.zip › nutrients-4229100-supplementary/Supplementary Table S2.pdf]

Supplementary Table S2. Key characteristics and findings of clinical studies investigating the use of prebiotics in patients with chronic kidney disease (CKD).

Type of prebiotic	Prebiotic dose	Study design	Patients characteristics	Sample Size	CKD stage	Dyalisis status	Method of administration Intervention Duration	Metabolic otcomes	Microbiota outcomes	Origin	Reference
Inulin + pea hull	10-15 g	Single-blind, pilot	62-68 years; eGFR: <50 mL/minute/1.73 m²	13	3-5		**Administration**: Oral (2 weeks: control (muffin + 5.5 g sucrose); 4 weeks: muffin with 10 g/day pea hull fiber; 6 weeks: muffin with 10 g/day pea hull fiber + 15 g of inulin) **Duration**: 12 weeks	↓↓ pCS	N/A	North America (Florida)	[61]
Arabinoxylan oligosaccharides (AXOS)	10 g	Placebo-controlled, double-blind, cross-over RCT	>18 years; eGFR: 15-45 mL/min/1.73 m²	40	3-4		**Administration**: oral (twice daily) **Duration**: 12 weeks (4 weeks AXOS and placebo, 4 weeks of wash-out treatment, then 4 weeks placebo and AXOS)	↓ TMAO<br>↔ pCS, IS, pCG, PAGln, HOMA-IR, insulin and glucose	N/A	Europe (Belgium)	[62]
Lactulose	30 mL	RCT	45-71 years; eGFR: ≤60 mL/min/1.73 m²	32	3-4		**Administration**: oral (3 times/day) **Duration**: 8 weeks	↓↓ Cr<br>↔ BUN, Hb	↑ *Bifidobacterium* spp., *Lactobacillus* spp.	Asia (Iran)	[63]
High-amylose maize resistant starch type 2 (HAM-RS2)	16 g	Double-blind, placebo-controlled RCT	>18 years	43	5	H	**Administration**: oral (Cookies: 3 days/week, during dialysis; Powder: sachets (4 days/week, at home, on not dialysis days)) **Duration**: 4 weeks	↓ IS, IL-6, TBARS	N/A	South America (Brazil)	[64]
Fructooligosaccharide (FOS)	3-12 g	Double-blind, placebo-controlled RCT	18-80 years; eGFR: <45 mL/min/1.73 m²	50	3–5		**Administration**: oral (initial dose: 3 g/day; increase by 3 g every 3 days, up to 12 g/day, twice/day) **Duration**:12 weeks	↓ HDL-C, tpCS, fpCS (in NDD-CKD patients)<br>↔ IS, IAA, fpCS	N/A	South America (Brazil)	[65]

High-amylose maize resistant starch type 2 (HAM-RS2)	20-25 g	Double-blind, placebo-controlled RCT	42-66 years	20	5	H	**Administration**: oral (20 g/day (during the first 4 weeks) and 25 g/day (during the second 4 weeks)) **Duration**:8 weeks	↓ BUN, IL-6, TNF-α, MDA	↑ *Faecalibacterium* genus	Asia (Iran)	[66]
High-amylose maize resistant starch type 2 (HAM-RS2)	20-25 g	Double-blind, placebo-controlled RCT	43-70 years	50	5	H	**Administration**: oral (20 g/ day (the first 4 weeks) and 25 g/day (the second 4 weeks)) **Duration**:8 weeks	↓ Cr, UA, pCS	N/A	Asia (Iran)	[67]
Inulin	19 g	Interventional, prospective and controlled	18-80 years; eGFR: 15-60 mL/min/1.73 m²	16	3-4		**Administration**: oral (once a day) **Duration**:24 weeks	↓ UA, TNF-α, CRP, NOX2 ↑ serum bicarbonate	↑ Bifidobacteriaceae ↓ Enterobacteriacae	Europe (Italy)	[68]
High-amylose maize resistant starch type 2 (HAM-RS2)	16 g	Double-blind, placebo-controlled RCT	45-65 years; BMI: 25.9 ± 5.42 kg/m²	16	5	H	**Administration**: oral (Cookies:1 package/day, during dialysis; Powder:1 sachet on not dialysis day) **Duration**: 4 weeks	↓ RANTES, PDGF-BB, IP-10	N/A	South America (Brazil)	[69]
Inulin-type fructans (ITFs) (inulin+FOS)	10 g	Double-blind, placebo-controlled, crossover RCT	18-65 years; BMI: 20.11 ± 2.80 kg/m²	21	5	CAPD	**Administration**: oral (once a day) **Duration**: 36 weeks (12 weeks inulin+FOS and placebo, 12 weeks of wash-out treatment, then 12 weeks placebo and inulin+FOS)	↓ faecal indole and pH ↓ IS (not significant) ↔ pCS	↓ *Bacteroides thetaiotaomicron*	Asia (China)	[70]
Inulin	19 g	Interventional, prospective and controlled	49-74 years; eGFR: 15-60 mL/min/1.73 m²	41	3-4		**Administration**: oral (once a day) **Duration**: 24 weeks	↓ insulin, glucose, HOMA-IR, TC, TG, HCY, UA, serum phosphorus and nitrogen ↑ serum bicarbonate, HDL-C	N/A	Europe (Italy)	[59]

Fructooligosaccharide (FOS)	6 g	Double-blind, placebo-controlled RCT	43-72 years; eGFR: 15-45 mL/min/1.73 m²	50	3-5		**Administration**: oral (twice daily)<br>**Duration**:12 weeks	↓ HDL-C, ionised calcium, albumin, ALP, IL-6<br>↑ sodium and bicarbonate	N/A	South America (Brazil) USA	[71]
β-Glucan	13,5 g	Single-blind, controlled RCT	48-72 years; eGFR: <60 mL/min per 1.73 m²	59	3-5		**Administration**: oral (once a day)<br>**Duration**:14 weeks	↓ fIS, fpCS<br>↓ tpCG/fpCG<br>↓ LDL-C (8 weeks), but at week 14 the difference disappeared)	*Faecalibacterium, Prevotella, Bacteroides, Blautia* and *Roseburia*= most abundant genera	South Africa(Cape Town)	[25]
Inulin-type prebiotics (inulin+FOS)	10 g	Double-blind, placebo-controlled, crossover RCT	26-49 years	33	5	CAPD	**Administration**: oral (once a day)<br>**Duration**: 36 weeks (12 weeks inulin+FOS and placebo, 12 weeks of wash-out treatment, then 4 weeks placebo and inulin+FOS)	↓↓UA<br>↑ fecal UA degradation<br>↔ purine intake or activity of XO	↓*Bacteroides intestinalis*<br>↑↑ ratio Firmicutes/Bacteroidetes<br>↑*Clostridium* spp. FS41, *Clostridium* spp. CAG:7, *Clostridium citroniae, Clostridium botulinum, Anaerostipes* spp. 3_2_56FAA, *Anaerostipes caccae*	Asia (China)	[72]
Oligofructose-enriched inulin (inulin+FOS)	8 g	Non-randomized, open-label 3-phase pilot	53-76 years; eGFR: 15-50 mL/min per 1.73 m²	17	3-4		**Administration**: oral (twice daily)<br>**Duration**: 12 weeks	↑ raffinose, L-Gln, 1-kestose, beta-gentiobiose, BS, allantoic acid, 4-MC (urine)	↓*Lachnospira, Moryella, Negativibacillus* genera<br>↓Ruminococcaceae, Erysipelotrichaceae<br>↑ *Bifidobacterium, Anaerostipes* genera	North America (USA)	[73]
Inulin	10 g	Double-blind, parallel RCT	51-65 years; eGFR: ≤ 44 mL/min/1.73 m²	54	3-5		**Administration**: oral (once a day)<br>**Duration**: 12 weeks	↓ IS, pCS<br>↓↓ IL-6<br>↔ Cr, BUN	N/A	Asia (China)	[60]

Oligofructose-enriched inulin (inulin+FOS)	8 g	Non-randomized, open-label, 3-phase pilot	57-80 years; eGFR: 15-50 ml/min per 1.73 m²	13	3-4	**Administration**: oral (twice daily)<br>**Duration**: 28 weeks	↓ urea cycle, adenine and UA, IS/pCS (urine), sucrose, glucose, fucose (post-treatment)<br>↓↓ DCA (faeces, urine and plasma)<br>↑ UFAs, phospholipid remodeling, PC acyl editing, glyoxylate cycle, FA degradation (post-treatment)<br>↑ Arg, ornithine, Lys, Thr, Met, Ile, Ala, Ser, and Gly biosynthesis, purine nucleotide degradation (post-treatment), xanthine, inosine, urate (faeces), L-glutamine, uridine (urine)	↑ *Bifidobacterium adolescentis, Bifidobacterium longum,* Lachnospiraceae (during treatment)<br>↑*Adlercreutzia equolifaciens, Clostridium bartlettii* (post-treatment)	North America (USA)	[74]

Abbreviations: RCT, randomized controlled trial; H, hemodialysis; CAPD, continuous ambulatory peritoneal dialysis; p-CS, p-cresyl sulfate; TMAO, trimethylamine N-oxide; IS, indoxyl sulfate; p-CG, p-cresyl glucuronide; PAGln, phenylacetylglutamine; HOMA-IR, homeostatic model assessment for insulin resistance; BUN, blood urea nitrogen; Cr, creatinine; IL-6, interleukin-6; Hb, hemoglobin; TBARS, thiobarbituric acid reactive substances; HDL-C, high-density lipoprotein cholesterol; tp-CS, total p-cresyl sulfate; fp-CS, free p-cresyl sulfate; NDD-CKD patients, non-dialysis-dependent chronic kidney disease patients; IAA, indole-3-acetic acid; TNF-α, tumor necrosis factor-alpha; MDA, malondialdehyde; CRP, C-reactive protein; UA, uric acid; NOX2, NADPH oxidase 2; RANTES, regulated upon activation, normal T-cell expressed and secreted; PDGF-BB, platelet-derived growth factor BB; IP-10, interferon-inducible protein 10; TC, total cholesterol; TG, triglycerides; HCY, homocysteine; ALP, alkaline phosphatase; fIS, free indoxyl sulfate; tp-CG, total p-cresyl glucuronide; fp-CG, free p-cresyl glucuronide; LDL-C, low-density lipoprotein cholesterol; XO, xanthine oxidase; L-Gln, L-glutamine; BS, beta-sitosterol; 4-MC 4-methylcatechol; DCA, deoxycholic acid; UFAs, unsaturated fatty acids; FA, fatty acids; PC, phosphatidylcholine; Arg, arginine; Lys, lysine; Thr, threonine; Met, methionine; Ile, isoleucine; Ala, alanine; Ser, serine; Gly, glycine; ↓, decreased; ↑, increased; ↔, unchanged; N/A, not available.
