# Supplementary material for: Impact of Probiotics, Prebiotics and Synbiotics Supplementation in Chronic Kidney Disease: A Comprehensive Review of Clinical Trials
Source: Nutrients. 2026 Apr 8;18(8):1176. doi: 10.3390/nu18081176 (PMC13118840; doi:10.3390/nu18081176)
Supplement: Supplementary file 1 [file nutrients-18-01176-s001.zip › nutrients-4229100-supplementary/Supplementary Table S3.pdf]

Supplementary Table S3. Key characteristics and findings of clinical studies investigating the use of synbiotics in patients with chronic kidney disease (CKD).

Synbiotic composition	Probiotics and Prebiotic dose	Study design	Patients characteristics	Simple Size	CKD stage	Dyalisis status	Method of administration Intervention duration	Metabolic outcomes	Microbiota outcomes	Origin	Reference
Lactobacillus acidophilus NCFM, *Bifidobacterium lactis* Bi-07, inulin	Probiotics:1,1x 10^7 CFU Prebiotic:2,31 g	Double-blind, placebo-controlled RCT	>18 years	42	5	H	**Administration**: oral (1 gel/day) **Duration**: 8 weeks	↓ CRP ↑ sodium, LDL-C, HDL-C ↔ IL-6, TNF-α	N/A	North America (Mexico)	[80]
Lactobacillus, *Bifidobacterium*, *Streptococcus* species, inulin, FOS, GOS	Probiotics:4,5 × 10^{10} CFU Prebiotics:7,5-15 g	Double-blind, placebo-controlled crossover RCT	59-79 years; eGFR: 10–30 mL/min71.73 m²	37	4-5		**Administration**: oral (Initial dose: 7.5 g of prebiotic and 1 probiotic capsule for 3 weeks; Full dose: 2 probiotic capsules/day + 15 g/day of prebiotic) **Duration**: 16 weeks (6 weeks synbiotic and placebo, 4 weeks of wash-out treatment, then 6 weeks placebo and synbiotic)	↓ pCS ↑ albuminuria ↔ eGFR proteinuria, IL-1β, IL-6, IL-10, TNF-α, LPS	↓ *Clostridiales* ↓ *Ruminococcaceae* ↑ *Bifidobacterium* spp. ↑ *Lachnospiraceae*	Oceania (Australia)	[26]
Lacticaseibacillus casei, Lactobacilus acidophilus, Lactobacillus delbrueckii subsp. *bulgaricus, Lacticaseibacillus rhamnosus, Streptococus thermophilus, Bifidobacterium breve, Bifidobacterium longum,* FOS	Probiotics:4,5 × 10^9 CFU Prebiotics:0,5 g	Double-blind, placebo-controlled RCT	35-75 years; eGFR:15–59 mL/min/1.73 m²	75	3-4		**Administration**: oral (2 g/day) **Duration**: 24 weeks	↓ BUN	N/A	Asia (Iran)	[55]

Lactobacillus acidophilus, *Bifidobacterium longum*, FOS	Probiotics + Prebiotics: 2 g	N/A	>18 years	26	2-5		**Administration**: oral (2 g/day)<br>**Duration**: 16 weeks	↑fIS, tIS, fpCS, tpCS<br>↓ PTH<br>↔ calcium	N/A	Europe (Italy)	[81]
Lactobacillus acidophilus, Bifidobacterium longum, Streptococcus thermophilus, FOS	Probiotics:0.1-0.4 g<br>Prebiotics: 0.1 g	Prospective, open labeled, interventional	>18 years	48	5	H	**Administration**: oral (1g sachet/twice daily)<br>**Duration**: 6 weeks	↓ hsCRP, TNF-α, IL-6,<br>↓ BUN, sodium, potassium, TG, TC, LDL-C<br>↑ GFR, Hb, Hct, ferritin, HDL-C	N/A	Asia (India)	[82]
Lactobacillus acidophilus, Bifidobacterium bifidum, Bifidobacterium lactis, Bifidobacterium longum, inulin, FOS, GOS	Probiotics: 2.7 × 10^7 CFU/g each<br>Prebiotics: 15 g	Double-blind, placebo-controlled RCT	30-65 years	50	5	H	**Administration**: oral<br>(5 g probiotic powder + 15g prebiotics, 4 times/day)<br>**Duration**: 12 weeks	↓↓ ICAM-1, VCAM-1<br>↔ CK-18, UA, phosphorus	N/A	Asia (Iran)	[43]
Lactobacillus coagulans, FOS	Probiotics + Prebiotics: 0.1 g	Double-blind, placebo-controlled RCT	46-80 years	50	5	H	**Administration**: oral (2 tablets/day)<br>**Duration**: 8 weeks	↓↓ hs-CRP, MDA<br>↓ TC, LDL-C<br>↔ TG, HDL-C	N/A	Asia (Iran)	[83]
Lactobacillus acidophilus, Bifidobacterium bifidum, Bifidobacterium lactis, Bifidobacterium longum, inulin, FOS, GOS	Probiotics: 2.7 × 10^7 CFU/g each<br>Prebiotics: 15 g	Double-blind, placebo-controlled RCT	30-65 years	50	5	H	**Administration**: oral<br>(5 g probiotic powder + 15g prebiotics, 4 times/day)<br>**Duration**: 12 weeks	↓↓ anti-HSP70, hs-CRP<br>↓ IL-6, endotoxins	N/A	Asia (Iran)	[44]

Lacticaseibacillus casei, Lactobacillus acidophilus, Lacticaseibacillus rhamnosus, Lactobacillus delbrueckii subsp. Bulgaricus, Bifidobacterium breve, Bifidobacterium longum, Streptococcus thermophilus, FOS	Probiotics:1×10^{10} CFU<br>Synbiotics: 0,5 g	Double-blind, placebo-controlled RCT	29-91 years	48	5	H	**Administration**: oral (2 capsules/day)<br>**Duration**: 8 weeks	↑ sodium<br>↑↑ PTH, albumin, calcium, IS<br>↓ phenol<br>↔ BUN, Cr	N/A	Asia (Iran)	[84]
Lacticaseibacillus casei LC4P1, *Bifidobacterium animalis* BLC1, FOS, inulin	Probiotics: 2,4 × 10^{9} CFU<br>Prebiotics: 5 g	Single-blind, placebo-controlled, pilot RCT	30–65 years; BMI: 18.5-29.9 kg/m²; eGFR: 21-34 mL/min/1.73 m²	23	3-4		**Administration**: oral (2 sachets/day)<br>**Duration**: 8 weeks	↓ BUN, calcium-phosphorus product, fIS<br>↑ sodium<br>↔ CRP, IL-6	N/A	Europe (Italy)	[85]
Lactobacillus acidophilus,Lactiplantibacillus plantarum,Lacticaseibacillus paracasei, L. delbrueckii subsp. bulgaricus, Bifidobacterium breve, Bifidobacterium longum, Bifidobacterium infantis, Streptococcus	Probiotics:4.5 × 10^{11} CFU<br>Prebiotics:10-20 g	Double-blind, placebo-controlled RCT	≥18 years; eGFR: 15-60 mL/min/1.73 m²	68	3-4		**Administration**: oral (½ dose/day (first 2 weeks), of 10 g of prebiotic powder and 1 probiotic sachet, then 20 g/day of prebiotic powder)<br>**Duration**: 48 weeks	↓ eGFR<br>↑ Cr	↑ *Bifidobacterium animalis*<br>↑ *Blautia* spp.	Oceania (Australia)	[27]

thermophilus, HRS											
Lactobacillus acidophilus, Bifidobacterium longum, *Streptococcus thermophilus*, FOS	Probiotics: 0.05 g - 0.1 g<br>Prebiotics: 0.1 g	Double-blind, placebo-controlled RCT	18–70 years; eGFR: <45 mL/min/1.73 m²	85	3-5		**Administration**: oral (3 capsules/day)<br>**Duration**: 12 weeks	↓ pCS, IS<br>↔ eGFR	N/A	Asia (India)	[86]
Lactobacillus acidophilus, Bifidobacterium longum, FOS	Probiotics:5x10^9 CFU<br>Prebiotics:0.06 g	Double-blind, placebo-controlled RCT with a parallel design	>50 years	60	5	H	**Administration**: oral (2 capsules/day)<br>**Duration**: 8 weeks	↔ IS	N/A	Asia (Indonesia)	[87]
Lacticaseibacillus casei LC4P1, *Bifidobacterium animalis* BLC1, FOS, inulin	Probiotics: 10^9 CFU<br>Prebiotics: 5 g	Single-blind, placebo-controlled, pilot RCT	30–65 years; BMI 18.5-29.9 kg/m²	23	3-4		**Administration**: oral (2 sachets/day)<br>**Duration**: 12 weeks (8 weeks treatment, then 4 weeks wash-out)	N/A	↓ *Flavobacteriaceae*<br>↑ *Coriobacteriaceae*<br>↑*Lachnospiraceae* (*Blautia* genus)<br>↑*Selenomonas* genus<br><u>Fecal VOCs:</u><br>↓ DMDS, trisulfide, NA, proteolytic metabolism<br>↑ 3-carene, AA, PA, 2-tridecanone, decane, saccharolytic metabolism	Europe (Italy)	[88]
Lactobacillus acidophilus, Lacticaseibacillus casei, Bifidobacterium lactis, inulin	Probiotics:8×10^9 CFU<br>Prebiotics:1,6 g	Double-blind, placebo-controlled RCT	≥18 years; eGFR:15-45 mL/min/1.73 m²	34	3-4		**Administration**: oral (2 probiotic+prebiotic capsules/day)<br>**Duration**: 12 weeks	↓↓ IS, hsCRP<br>↓ p-CS<br>↑ eGFR	↑ *Bifidobacteria, Lactobacillus, Subdoligranulum* genera	Europe (Serbia)	[89]
Lactobacillus acidophilus La-14, FOS	Probiotics: 2 × 10^{11} CFU<br>Prebiotics: 0,065 g	Prospective, quasi-experimental single-center	>18 years; eGFR: 10-22 mL/min/1.73 m²	30	5	H	**Administration**: oral (once a day)<br>**Duration**: 8 weeks	↓↓IS, pCS<br>↓MDA, IL-6	N/A	Europe (Bulgaria)	[90]

Lactobacillus acidophilus La-14, FOS	Probiotics: 2 × 10^{11} CFU<br>Prebiotics: 0,065 g	Prospective, single-blind, non-RCT, placebo-controlled	46-70 years	50	4-5	H	**Administration**: oral (1 capsule/day)<br>**Duration**: 40 weeks	↓↓ IS<br>↔ p-CS, IL-6, MDA, eGFR	N/A	Europe (Bulgaria)	[91]

Abbreviations: RCT, randomized controlled trial; H, hemodialysis; CRP, C-reactive protein; LDL-C, low-density lipoprotein cholesterol; HDL-C, High-Density Lipoprotein Cholesterol; IL-6, interleukin-6; TNF-α, tumor necrosis factor-alpha; IL-10, interleukin-10; p-CS, p-cresyl sulfate; BUN, blood urea nitrogen; LPS, lipopolysaccharide; eGFR, estimated glomerular filtration rate; IL-1β, interleukin-1 beta; fIS, free indoxyl sulfate; tIS, total indoxyl sulfate; tp-CS, total p-cresyl sulfate; fp-CS, free p-cresyl sulfate; hs-CRP, high-sensitivity C-reactive protein; TC, total cholesterol; TG, triglycerides; GFR, glomerular filtration rate; ICAM-1, intercellular adhesion molecule-1; Hct, hematocrit; Hb, hemoglobin; V-CAM-1, vascular cell adhesion molecule-1; MDA, malondialdehyde; Cr, creatinine; eGFR, estimated glomerular filtration rate; CK-18, cytokeratin-18; UA, uric acid; anti-HSP70, anti-heat shock protein 70; VOCs, volatile organic compounds, DMDS, dimethyl-disulfide; PA, propionic acids; AA, acetic acid; NA, nonanoic acid; ↓, decreased; ↑, increased; ↔, unchanged; N/A, not available.
